# Regulating Droplet Dynamics via Wormlike Micelle Networks: From Splash Suppression to Precise Confined Spreading

**DOI:** 10.1002/advs.76684

**Published:** 2026-07-29

**Authors:** Shuling Liu, Beibei Xie, Xingyue Lou, Yu Deng, Lei Wang, Jiawei Li, Dongming Qi

**Affiliations:** ^1^ Engineering Research Center For Eco‐Dyeing and Finishing of Textiles Zhejiang Sci‐Tech University Hangzhou Zhejiang China; ^2^ Key Laboratory of Advanced Textile Materials and Manufacturing Technology and Engineering Research Center For Eco‐Dyeing & Finishing of Textiles, Ministry of Education Zhejiang Sci‐Tech University Hangzhou Zhejiang China; ^3^ Zhejiang Provincial Innovation Center of Advanced Textile Technology Shaoxing Zhejiang China

**Keywords:** cationic compounds, cellulose, droplet impact, wormlike micelle

## Abstract

The high‐speed impact of droplets is fundamental to frontier applications ranging from high‐resolution inkjet printing to precision agriculture. However, simultaneously suppressing stochastic splashing and achieving controllable, uniform spreading on hydrophilic surfaces remains a formidable challenge. Herein, we report a strategy to modulate droplet dynamics, which triggers a structural transition in anionic surfactant (SDS) aggregates via quaternary ammonium cationic induction (CT_1_, CT_4_). This transition yields a sophisticated internal wormlike micelle (WLM) network that facilitates splash‐free, spatially confined deposition. As a proof of concept, we demonstrate the dual‐regulatory role of the WLM network during high‐speed impact on cotton fabrics. Specifically, the synergistic interaction between CT_4_‐SDS aggregates and the ionic bonding with the cellulose backbone effectively inhibits excessive spreading during the initial wetting phase. Furthermore, the entanglement of the WLM network with the hierarchical micro/nano‐structures of cellulose fibers generates a powerful interfacial mechanical pinning effect. This effect immobilizes the three‐phase contact line and constructs a spatial barrier at the spreading front. As a result, a stabilized liquid film and uniform circular deposition are ensured. Overall, this study provides a novel mechanistic pathway for steering droplet impact kinetics and offers critical insights into the design of complex fluids for high‐performance functional coating and deposition.

## Graphical abstract

As displayed in Graphical Abstract image.tif, a molecular‐design strategy precisely tailors high‐speed droplet dynamics through programmed surfactant co‐assembly. The structural transition into an entangled wormlike micelle network completely suppresses stochastic splashing on fibrous substrates. This internal architecture drives rapid interfacial activity and powerful mechanical pinning, unlocking a universal paradigm for high‐precision fluid patterning and deposition.

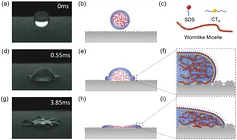

## 1 Introduction

The high‐speed impact of inertial droplets on substrates plays a critical role in many applications. These applications demand precise deposition and uniform morphology, such as inkjet printing [1, 2], biochip array design [3], fabrication of high‐performance electronic circuits [4], and targeted pesticide spraying [5, 6]. Extensive research has been devoted to understanding the dynamics of droplet impact, with significant advances in suppressing splashing and rebound [7, 8]. A promising pathway to control droplet behavior leverages surfactant self‐assembly, where molecules form diverse microstructures—including vesicles [9, 10, 11], spherical micelles [12], rod‐like micelles [13], and wormlike micelles [14, 15, 16]. The formation and evolution of these assemblies are fundamentally governed by the molecular structure of the constituent compounds. For cationic species, the strongly positively charged groups can engage in electrostatic interactions and form ionic bonds with anionic surfactants, thereby directing a structural transition of the surfactant aggregates [17, 18, 19, 20]. Consequently, an internal micellar network forms within the droplet. This network modifies the rheological and surface tension properties of the system [21], effectively suppressing splashing and rebound while promoting uniform spreading [22].

Although the addition of surfactants can enhance droplet spreading by reducing surface tension [23], such a reduction may also increase impact instability. In some cases, it can even trigger droplet splashing and satellite droplet [24] formation via Kelvin‐Helmholtz instability [25]. The maximum wave number associated with this instability is given by k_max_∼2ρ_a_Ur^2^/3γ, where ρ_a_ is air density. Therefore, achieving uniform spreading on hydrophilic surfaces without inducing splashing remains a key challenge.

Recent strategies for controlling droplet impact outcomes fall into two broad categories. One modifies the liquid's intrinsic properties, while the other alters external conditions. Internally, structural modifications of the impacting liquid have proven effective. For instance, Jiang et al. demonstrated that electrostatic binding between triamine and SDS reduces charge repulsion and enhances hydrophobic interactions. At a molar ratio R = 0.5 (triamine to SDS), a dense wormlike micelle network forms. This network increases droplet viscosity and reduces surface tension, thereby suppressing splashing and promoting uniform spreading even on superhydrophobic surfaces [22]. Similarly, Klein Schaarsberg et al. suppressed splashing in suspension droplets by increasing the solvent viscosity to raise the Stokes number while reducing the particle Weber number to limit kinetic energy [26]. Beyond intrinsic properties, external fields offer a highly tunable means of control. One approach is modifying the substrate. Lee et al., for example, used microporous fabric structures to pin droplets via capillary pressure, thereby suppressing rebound [27]. Hamdan et al. promoted the formation of finer secondary droplets above a threshold Weber number during impact on a Leidenfrost‐temperature hot surface, enabling controlled splashing refinement [28]. Other approaches involve applying external fields. For instance, Yu et al. applied controlled charging to generate electric stresses that counteract aerodynamic lift and suppress splashing [29]. Meanwhile, Ram Krishna et al. employed external magnetic fields to attract ferrofluid droplets, effectively inhibiting rebound from hydrophobic surfaces in a tunable manner [30].

Despite these advances, current strategies exhibit notable limitations. For example, the use of external fields (electric, magnetic) requires complex setup and is difficult to integrate into continuous processes; environmental control methods (pressure, temperature) is often costly and lack generality. More importantly, many approaches fail to systematically connect molecular‐scale interactions, surfactant assembly evolution, macroscopic rheology and dynamic spreading behavior, resulting in limited predictive accuracy.

To address these critical challenges, we present a molecular‐design‐driven strategy to autonomously modulate droplet internal architectures, which also circumvents the need for complex external fields. We systematically investigate the synergistic co‐assembly of anionic surfactant (SDS) with tailored quaternary ammonium cations, including (3‐chloro‐2‐hydroxypropyl) trimethylammonium chloride (CT_1_) and its butane‐analogous counterpart (CT_4_). And we also study their resulting impact dynamics on cotton fabrics. By integrating rheological profiling with cryogenic transmission electron microscopy (Cryo‐TEM), we directly visualize the evolution of SDS aggregates. This evolution proceeds from discrete spheres to sophisticated wormlike micelle (WLM) networks, thereby establishing a robust structure–property‐function paradigm. Furthermore, we propose a comprehensive quantitative model correlating the maximum and stabilized spreading diameters with the dimensionless Reynolds (N_Re_) and Weber (N_We_) numbers. This work elucidates how the transient viscoelasticity of WLM networks, coupled with tailored interfacial tension dynamics, synergistically dissipates kinetic energy to suppress splashing and immobilize the spreading front. Our findings offer a universal pathway for high‐precision liquid deposition.

## 2 Results and Discussion

The impact behaviors of SDS and CT_n_/SDS droplets were recorded by a high‐speed camera at atmospheric pressure with a relatively high impact velocity of 1.98 m s^−1^ on the cotton surfaces. Figure 1 indicates the contrast spread dynamics of an SDS drop and CT_1_/SDS drops impacting cotton surfaces at an impacting velocity (U) of 1.98 m s^−1^ from side views (Movie S1). The diameter (D_0_) of SDS and CT_1_/SDS droplets is about 3.64 and 3.60–3.63 mm, respectively.

**FIGURE 1:**
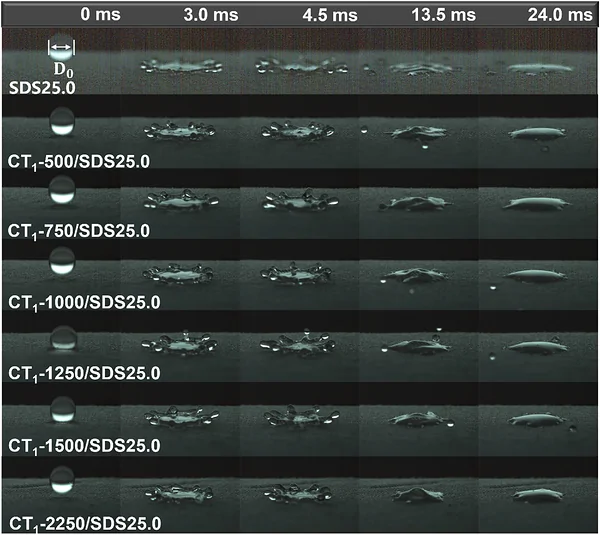
Dynamical behavior of impacting SDS and CT_1_/SDS25.0 drops with different CT_1_ concentrations on the cotton fabric.

Upon impact of the SDS solution droplet with cotton fabric, the collision dynamics progress through three distinct regimes, including the kinetic‐dominated phase (K‐phase), relaxation phase (R‐phase), and capillary‐driven wetting phase (W‐phase). The K‐phase is dominated by the high kinetic energy of the droplet and splash generally occurs; the K‐phase begins with the droplet's contact with the cotton to 3.0 ms (Figure 1). Subsequently, the system enters the R‐phase. Here, energy dissipation through viscous damping induces oscillatory deformation of the primary droplet while maintaining a relatively steady spreading diameter. The process culminates in the W‐phase as capillary forces dominate, achieving maximum liquid‐substrate contact area through spontaneous imbibition into the fibrous network. Remarkably, the CT_1_/SDS composite droplets formed via electrostatic pairing between the quaternary ammonium moieties of CT_1_ and the sulfate groups of SDS exhibit restricted spreading dynamics and suppressed splashing instabilities, originating from enhanced droplet viscoelasticity. The enhancement is induced by reorganization within SDS surfactant assemblies.

To investigate the influence of CT_1_ concentration on the impact dynamic behavior of CT_1_/SDS droplets, experiments are conducted with fixed SDS concentration (25.0 mM) and varying CT_1_ concentrations (0–2250 mM). As illustrated in Figure 1, the incorporation of CT_1_ significantly suppresses droplet splashing. Compared to SDS droplets without CT_1_, CT_1_/SDS droplets exhibits markedly reduced splashing phenomena at 25.0 mM SDS. Notably, when CT_1_ concentration increases to 2250 mM, no droplet splashing is observed on cotton fabric surfaces. Simultaneously, the spreading performance β (β = D_t_/D_0_, where D_0_ denotes the initial droplet diameter and D_t_ represents the instantaneous spreading diameter) of CT_1_/SDS droplets progressively declines as CT_1_ concentration increases from 500 to 2250 mM under fixed SDS concentration (25.0 mM). This β value remains substantially lower than that of SDS droplets without CT_1_, indicating a concentration‐dependent inhibitory effect of CT_1_ on both splashing behavior and spreading dynamics. These results demonstrate that the synergistic interaction between SDS and CT_1_ critically governs droplet impact stability and interfacial spreading efficiency on cotton fabric.

The CT_1_/SDS drops with different SDS concentrations are constructed to investigate the effect of SDS micelles on the droplet impacting dynamic stability. As shown in Figure 2, under a fixed SDS:CT_1_ molar ratio of 1:30, the splashing phenomena of CT_1_/SDS droplets ‌are observed to diminish significantly as SDS concentration increases from 25.0 to 150 mM. Notably, at SDS concentrations exceeding 35.0 mM, droplet splashing ‌is fully suppressed during impacts with cotton fabrics. High‐speed imaging further ‌reveals that at SDS concentrations of 100 mM and 150 mM, no droplet detachment ‌is detected at the R‐phase. Concurrently, the dimensionless spreading factor β of CT_1_/SDS droplets demonstrates a concentration‐dependent attenuation across the 25.0–150 mM SDS concentration range at a fixed SDS:CT_1_ molar ratio of 1:30. These results ‌highlight a dual‐phase suppression mechanism: elevated SDS concentrations ‌enhance droplet viscoelasticity to inhibit splashing, while simultaneously restrict spreading dynamics. The CT_1_/SDS synergistic assembly ‌thus establishes tunable energy dissipation pathways for precision control of droplet impact outcomes on the cotton fabric.

**FIGURE 2:**
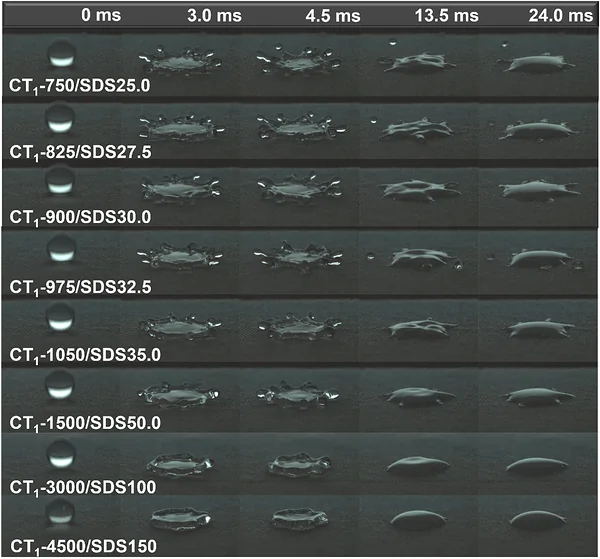
Dynamical behavior of impacting CT_1_/SDS drops with different SDS concentrations on the cotton fabric.

The SDS droplets with different CT_n_ are constructed to investigate the effect of CT structure on the droplet impacting dynamic stability (Figures S1 and S2). The viscosity, surface tension, and Re parameters of different types of CT_n_/SDS are shown in Table S1. The CT_4_‐175/SDS25.0, CT_4_‐250/SDS25.0, CT_4_‐200/SDS50.0, CT_4_‐400/SDS100, and CT_4_‐600/SDS150 indicate well‐spread behavior, restricted splash is observed and spreading behavior is better restrained during the whole impacting process (Movie S2). Compared to CT_1_, CT_4_‐based systems achieve superior splash suppression and spreading restraint, highlighting the critical role of the cationic alkyl chain length in modulating droplet impact dynamics.

In order to gain further insight into the distinctive characteristics of CT_n_/SDS droplet, we conducted a time‐resolved analysis of the normalized spread diameter (D_t_/D_0_) for all samples (Figure 3a,b). The CT_4_/SDS system exhibited superior performance in suppressing droplet splashing at significantly lower molar ratios compared to CT_1_/SDS, a distinction stemming from their inherent structural differences. This is evidenced by formulations such as CT_1_‐2250/SDS25.0, CT_4_‐250/SDS25.0, CT_1_‐1000‐4500/SDS35.0‐150, as well as CT_4_‐200‐600/SDS50.0‐150, all of which achieved uniform, splash‐free spreading. Figure 3c illustrates the α_f_ and β_f_ values of all samples with varying Re values on the cotton fabric. The CT_1_‐4500/SDS150 droplet (Re = 286) indicates the minimum α_f_ (1.81) and β_f_ (3.09) on the cotton fabric. As the Re values increase, α_f_ and β_f_ values increase accordingly, with the SDS25.0 droplet demonstrating the highest α_f_ (3.25) and β_f_ (6.87) values. The CT_4_/SDS system achieves splash suppression at much lower molar ratios than CT_1_/SDS, and higher Re numbers correlate with increased spreading parameters.

**FIGURE 3:**
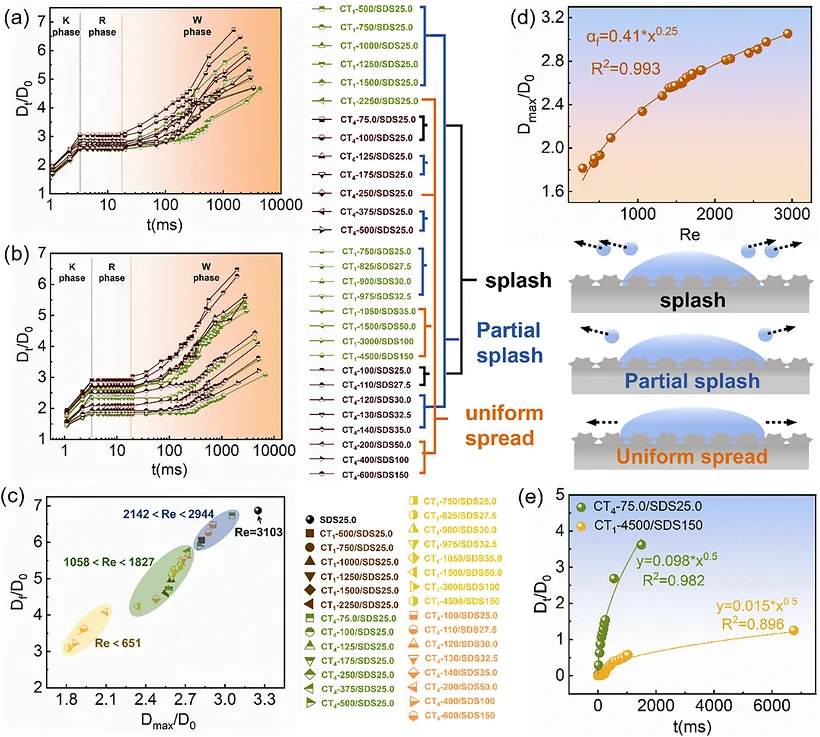
Impacting and spreading behavior of CT_n_/SDS droplet. (a) The spreading diameter as a function of time for CT_n_/SDS25.0 droplet. (b) The spreading diameter as a function of time for CT_n_/SDS25.0–150 droplet. (c) The α_f_ and β_f_ values of all samples with varying Re values on the untreated cotton. (d) Fitted curve of the α_f_ varies with Re. (e) Spreading diameter versus spreading time for CT_4_‐75.0/SDS25.0 and CT_1_‐4500/SDS150 droplet on the untreated cotton.

The maximum spreading parameter α_f_ during K‐phase, α_f_ = D_max_/D_0_, for different CT_n_/SDS systems is indicated in Figure S3, here, D_max_ refers to the maximum spreading diameter during K‐phase, D_0_ is the initial diameter. Notably, the CT_1_‐4500/SDS150 droplet exhibits minimal spreading behavior with α_f_≈1.81 on untreated cotton substrates‌. The CT_1_/SDS25.0 demonstrates a linear reduction in α_f_ from 2.82 to 2.55 as CT_1_ concentration increases from 500 to 2250 mM, while CT_4_/SDS25.0 displays non‐monotonic behavior with α_f_ decreasing from 3.05 to 2.57 (75.0–250 mM CT_4_) before increasing to 2.97 (250–750 mM CT_4_)‌. In systems maintaining fixed CT_n_/SDS molar ratios, CT_1_‐750‐4500/SDS25.0‐150 shows two‐phase variation. The α_f_ values remain stable within the CT_1_‐750‐975/SDS25.0‐32.5 concentration range (α_f_≈2.67 ± 0.05), followed by a significant decrease to 1.81 at maximum concentrations (CT_1_‐4500/SDS150). Similarly, CT_4_‐100‐600/SDS25.0‐150 systems exhibit primary reduction in α_f_ from 2.91 to 1.86 (CT_4_‐100‐400/SDS25.0‐100) with marginal rebound to 1.93 at maximum concentrations. The droplet spreading results reveal that synergistic regulation between SDS micellar aggregates and CT_n_ structure effectively suppresses droplet spreading kinetics across the K‐phase.

The final spreading parameter β_f_, β_f_ = D_f_/D_0_, which is proportional to the spreading efficiency, for different CT_n_/SDS system are indicated in Figure S4. In CT_1_/SDS25.0 systems, β_f_ undergoes a linear reduction from 6.06 to 4.61 with CT_1_ concentration escalation (500–2250 mM). Contrastingly, CT_4_/SDS25.0 systems exhibit biphasic behavior: β_f_ initially declines from 6.71 to 4.69 (75.0–250 mM CT_4_) before rebounding to 6.57 at 750 mM CT_4_. Fixed‐ratio CT_1_/SDS systems display threshold‐governed evolution where β_f_ stabilizes near 5.4 (750–975 mM CT_1_/25.0–32.5 mM SDS) followed by abrupt collapse to 3.09 at maximum concentrations. Similarly, fixed‐ratio CT_4_/SDS systems with varying SDS concentrations show primary β_f_ suppression from 6.47 to 3.22 (25.0–100 mM) before partial recovery to 3.63 at elevated SDS concentrations (150 mM).‌ The droplet spreading results reveal that synergistic regulation between SDS micellar aggregates and CT_n_ structure effectively suppresses droplet spreading kinetics across both K‐phase and W‐phase.

The spreading dynamics of droplets on cotton fabrics can be analyzed through energy conservation principles. Before impact, the kinetic energy (E_K1_) and surface energy (E_S1_) of the spherical drop are given by

(1) $$E_{K1}=\frac{1}{2}\left(\rho \frac{\pi }{6}D_{0}^{3}\right)V_{0}^{2}$$

(2) $$E_{S1}=\pi D_{0}^{2}\gamma$$

Here ρ denotes the droplet density, D_0_ is the initial diameter. V_0_ is the impact velocity, γ is the liquid‐air surface tension.

At maximum spreading diameter D_max_ during the K‐phase, the kinetic energy diminishes to zero, while the surface energy becomes:

(3) $$E_{S2}=\frac{\pi }{4}D_{\max}^{2}\gamma \left(1-\cos\theta_{a}\right)$$

Here θ represents the equilibrium contact angle at the gas‐liquid‐solid interface.

The energy dissipation through viscous resistance (W) is quantified as:

(4) $$W=\frac{\pi }{3}\rho V_{0}^{2}D_{0}D_{\max}^{2}\frac{1}{\sqrt{Re}}$$

Applying the energy conservation principle, E_K1_+E_S1_ = E_S2_+W, and combining Equation (1), (2), (3), and (4), we derive the maximum spread factor‌ during K‐phase:

(5) $$\alpha_{f}=\frac{D_{\max}}{D_{0}}=\sqrt{\frac{We+12}{3\left(1-\cos\theta_{a}\right)+4\left(We/\sqrt{Re}\right)}}$$

Here We = ρV^2^D_0_/γ denotes the Weber number, Re = ρVD_0_/µ is the Reynolds number.

Given the hydrophilic nature of cotton surfaces, the term (1−cosθ_a_) in Equations (5) approaches zero. When We≫12, Equation (5) simplifies to:

(6) $$\alpha_{f}\approx {Re}^{0.25}$$

Experimental validation through droplet impact measurements on cotton fabrics demonstrates strong correlation between the maximum spreading diameter α_f_ and Reynolds number, as shown in Figure 3d. The nonlinear regression model (R^2^ = 0.993) confirms that droplet dynamics during the K‐phase are governed by inertial‐viscous interactions, with excellent agreement between theoretical predictions and empirical data (see Equation 7).‌

(7) $$\alpha_{f}=0.41*Re^{0.25}$$

From the above, it can be seen that the maximum spreading factor α_f_ scales with Re^0.25^, and the energy‐conservation‐based model accurately predicts droplet spreading behavior on hydrophilic cotton fabrics.

Figure 4 elucidates the critical factors that govern the uniform spreading and deposition of liquid droplets. These factors are mediated by cationic oligomer‐induced surfactant assemblies, which form via strong electrostatic binding and hydrophobic interaction between SDS and CT_n_, from molecular and aggregate perspectives. To investigate the physicochemical factors dictating droplet impact behavior, we systematically varied the molar ratio R (CT_4_ to SDS) and the SDS concentration (C_SDS_). At a fixed C_SDS_ of 25.0 mM with no CT_4_ (R_CT4/SDS_ = 0), only small spherical micelles (∼2.2 nm) formed (Figure 4a), corresponding well with DLS measurements (Figure S5). With the increase of molar ratio R (CT_n_ to SDS) from 0 to 10, the CT_4/_SDS25.0 aggregate gradually changed from spherical to wormlike (Figures 4a,b). The surfactant aggregates progressively elongated, with a sharp increase in length observed beyond R_CT4/SDS_ = 5 (Figure 4c and Figure S6, for scattering intensity data [31] of the CT_4_/SDS system). At R_CT4/SDS_ = 7, dense and entangled networks of wormlike micelles formed (Figure 4b). These dense networks can undergo extension and alignment under applied shear, leading to enhanced local hydrodynamic viscosity. Consequently, the solution viscosity approximately doubled as R_CT4/SDS_ increased from 3 to 10 (Figure 4c). At R_CT4/SDS_ = 20, progressive disassembly of the entangled micellar network occurs, accompanied by fragmentation of wormlike structures. This microstructural degradation drives a corresponding viscosity reduction from 4.75 to 3.25 mPa·s as R_CT4/SDS_ increases from 10 to 20 (Figure 4c). Simultaneously, across the concentration gradient (C_SDS_ = 25.0–100 mM), proliferation of wormlike micellar assemblies occurs with increasing SDS concentration. The densely entangled micellar networks undergo shear‐induced alignment and extension, thereby enhancing local hydrodynamic viscosity. Consequently, with increasing C_SDS_ from 25.0 to 100 mM, the viscosity rises markedly from 2.85 to 16.92 mPa·s. Beyond optimal concentration (100 mM<C_SDS_<150 mM), progressive disassembly of entangled networks and fragmentation of wormlike micellar structures emerge. This microstructural degradation drives viscosity reduction from 16.92 to 14.23 mPa·s (Figure 4d). Within the CT_1_/SDS system at fixed C_SDS_ = 25.0 mM, increasing the R_CT1/SDS_ from 20 to 90 progressively enhances wormlike micelle formation, though with limited structural proliferation. This constrained microstructural evolution drives a correspondingly modest viscosity increase from 3.28 to 5.16 mPa·s (Figure S7). When modulating C_SDS_, viscosity remains nearly constant at 5 mPa·s between 25.0–35.0 mM, indicating a plateau phase. Beyond this threshold (35.0–150 mM), proliferation of wormlike micellar assemblies drives pronounced viscosity escalation from 4.61 to 25.21 mPa·s (Figure S8).

**FIGURE 4:**
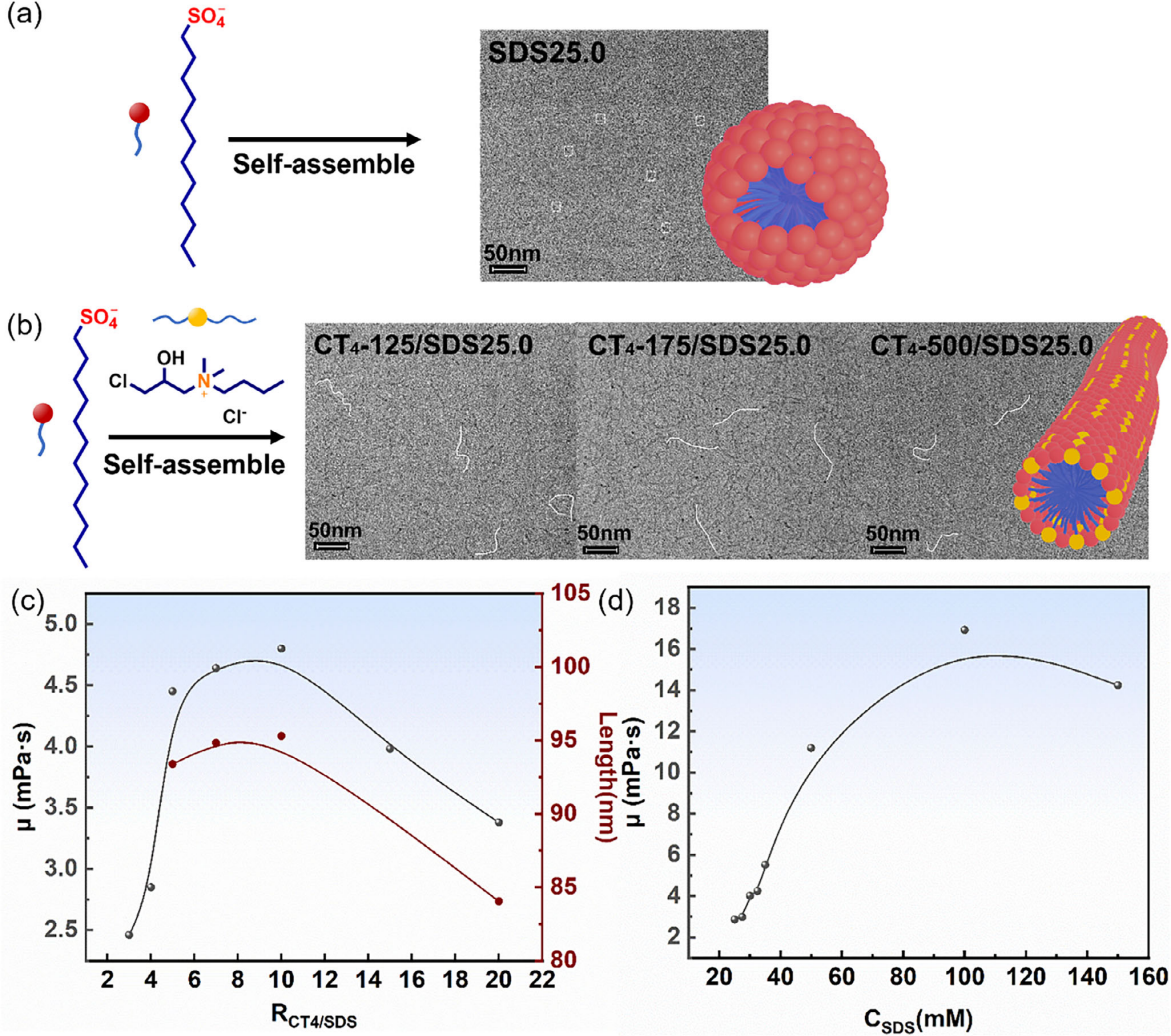
Morphological transition and rheological behavior of SDS and CT_4_/SDS aggregate systems. (a,b) Cryo‐TEM micrographs and corresponding schematics of surfactant aggregates: (a) SDS alone forms spherical micelles (≈2 nm diameter, dotted rectangles). (b) CT_4_/SDS complexes assemble into elongated, densely entangled wormlike micellar networks. (c,d) Evolution of solution viscosity and aggregate contour length as functions of molar ratio (R_CT4/SDS_) and SDS concentration (C_SDS_). Beyond critical thresholds, micellar elongation and network percolation induce pronounced shear‐thinning behavior.

Critically, at fixed C_SDS_ = 25.0 mM, CT_4_/SDS systems exhibit significantly enhanced viscosity responsiveness compared to CT_1_/SDS counterparts. While minimal CT_4_ addition induces substantial viscosity modulation, CT_1_/SDS solutions require substantially higher oligomer loading for comparable effects. Furthermore, equivalent viscosities emerge at elevated concentrations: both CT_4_‐130/SDS32.5 and CT_1_‐975/SDS32.5 systems reach≈4.2 mPa·s at 32.5 mM SDS, while CT_4_‐400/SDS100 and CT_1_‐3000/SDS100 both achieve≈16.7 mPa·s at 100 mM SDS. This equivalence occurs despite CT_4_ requiring merely 13.3% of CT_1_'s molar concentration, attributable to CT_4_'s extended alkyl chains, which enhances its interaction with SDS molecules compared to CT_1_. The stronger binding promotes a structural transition of SDS aggregates at a lower concentration, resulting in micelles with a higher aggregation number and an increased entanglement density. These changes collectively lead to the formation of a robust viscoelastic network. We further characterized the viscoelastic properties through measurements of storage (G') and loss (G″) moduli (Figure S9). The CT_4_/SDS solutions exhibited liquid‐like behavior (G'<G'') [32, 33]. Conclusively, bulk rheological profiling highlights that due to its extended hydrophobic tail, CT_4_ achieves equivalent viscoelastic network strength and a peak viscosity amplification of 16.92 mPa·s while consuming merely 13.3% of the molar concentration required by its CT_1_ counterpart before undergoing microstructural fragmentation at hyper‐optimal concentrations.

The cationic oligomer‐induced surfactant assemblies also exhibit superior surface tension reduction in aqueous solutions. Increasing the R_CT4/SDS_ ratio from 0 to 3 significantly lowered both the initial surface tension (from 52.25 to 43.67 mN·m^−1^) and the equilibrium surface tension (from 36.43 to 32.98 mN·m^−1^) (Figure 5a). This pronounced reduction suggests that the CT_4_/SDS assemblies rapidly adsorb and undergo interfacial jamming at the air/liquid interface, forming robust interfacial layers with exceptionally low surface tension. All CT_4_‐modified systems exhibit significantly reduced initial and equilibrium surface tensions relative to the R = 0 reference (pure SDS solution).

**FIGURE 5:**
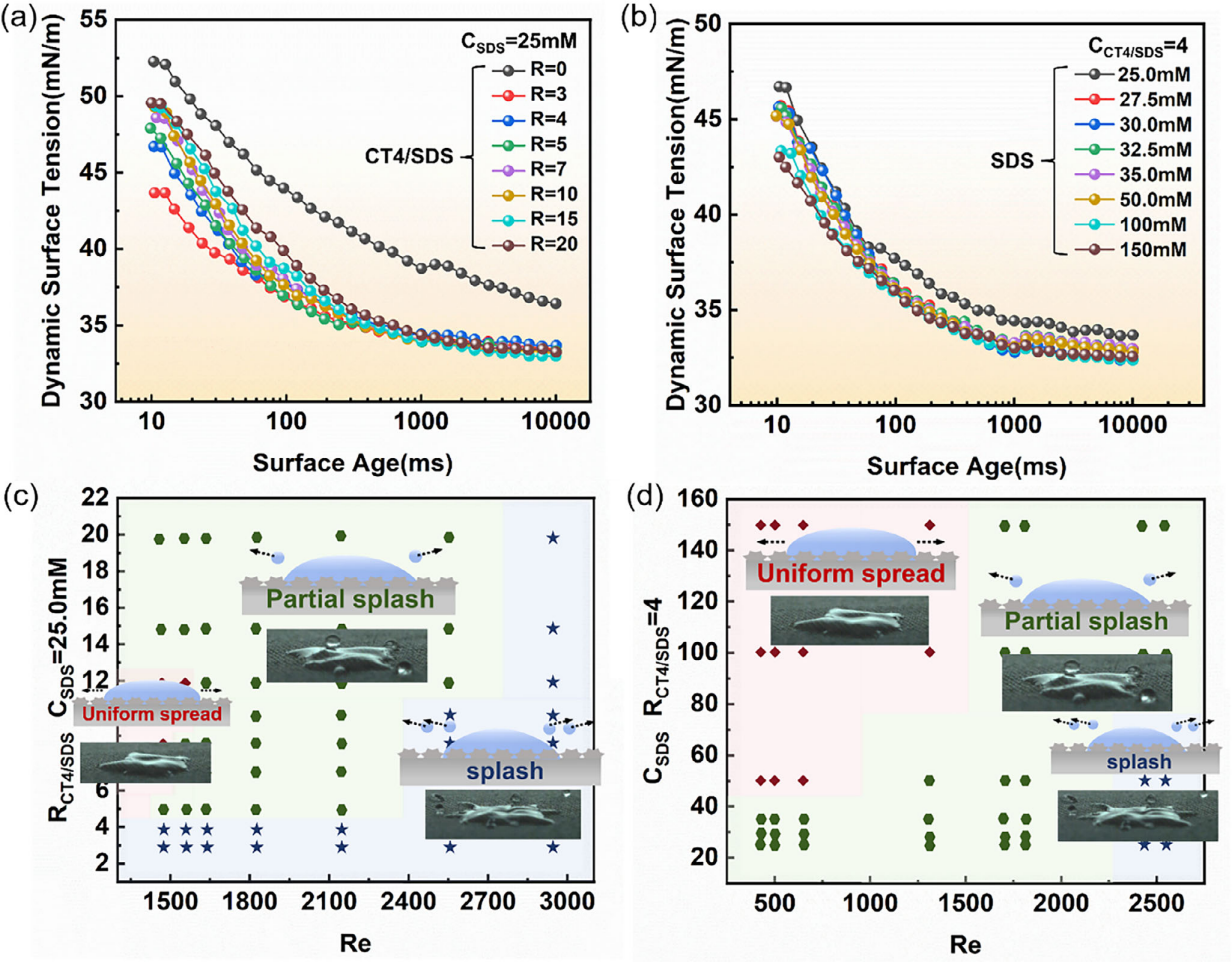
Dynamic surface tension and droplet impact phase behavior of SDS and CT_4_/SDS aggregate systems. (a,b) Dynamic surface tension profiles demonstrating significant reduction in both initial and equilibrium values with increasing R_CT4/SDS_ and C_SDS_. (c,d) Phase diagrams correlating droplet impact outcomes on superhydrophobic surfaces with Reynolds number (Re), revealing complete deposition occurs exclusively at low Re within optimal parameter range: R_CT4/SDS_ = 10–12 and 80.0 mM<C_SDS_<150 mM.

However, as observed in Figure 5c, droplet splashing persists within the R_CT4/SDS_ ratio range from 0 to 3. This behavior can be attributed to the predominance of spherical micelles and the absence of a well‐developed wormlike micellar network at this stage, resulting in an insignificant increase in solution viscosity. These findings further demonstrate that viscosity plays a dominant role in regulating droplet spreading behavior. Complete deposition and uniform spreading are achieved within the Reynolds number range of 1480<Re<1600 for R_CT4/SDS_ ratios between 5 and 12. However, distinct spreading morphologies are observed within this range. Specifically, drops exhibit complete and uniform spreading devoid of radial spokes at R_CT4/SDS_ ratios between 10 and 12. In contrast, radial spokes are present during spreading at lower ratios, specifically between 5 and 10. Notably, at R_CT4/SDS_ ratios further increases beyond the optimal range (12<R_CT4/SDS_<20), a marked degradation in deposition performance is observed. The uniform spreading regime gives way to splashing phenomena, characterized by droplet detachment and satellite droplet formation (Figure 5c and Movie S3). This trend is fully consistent with the experimental observations presented in Figure 4a–c. In detail, the structural evolution and quantity of SDS aggregates are modulated by the concentration of CT_4_. The Cryo‐TEM results demonstrate a clear microstructural evolution: at low ratios (R = 0–5), aggregates transition from spherical to wormlike micelles, leading to a substantial increase in viscosity that suppresses extensive splashing and promotes uniform spreading. Within the optimal range (R = 5–12), the system exhibits uniform spreading due to a well‐defined, percolating network of wormlike micelles. However, as the ratio increases further (R = 12–20), the micellar network is progressively disrupted, and the number of micelles decreases, resulting in a gradual reduction in viscosity. This loss of viscoelasticity causes the deposition behavior to revert from uniform spreading back to partial splashing.

The observed structural transition from spherical to wormlike micelles in the CT_4_/SDS system is reminiscent of the hybrid micellization reported in other cationic/anionic systems, such as CTAB‐SDS mixtures, where increasing the cationic surfactant concentration drives a consecutive transition from spherical micelles to wormlike micelles and ultimately to vesicles [34]. However, our CT_4_/SDS system exhibits a sharp viscosity increase at a much lower cationic‐to‐anionic molar ratio (R_CT4/SDS_ ≈ 5–10) compared to the AHSB/SDS system reported by Lu et al., which required higher surfactant loading and additional salt to achieve comparable viscosity enhancement [35]. This superior efficiency is attributed to the stronger electrostatic binding of CT_4_'s quaternary ammonium headgroup and the favorable hydrophobic matching between CT_4_'s butyl chain and SDS's dodecyl chain.

Uniform droplet spreading further requires sufficiently high CT_4_/SDS concentrations. At R_CT4/SDS_ = 4, uniform spreading (Re = 1 300) is achieved at C_SDS_ between 80.0 and 150 mM (Figure 5d and Movie S4). With the molar ratio of CT_4_ to SDS kept constant, an increase in SDS concentration from 25.0 to 100 mM promotes the formation of a larger number of SDS aggregates. This rise in aggregate prevalence subsequently alters both the droplet viscosity and surface tension. The viscosity at C_SDS_ = 100 mM is approximately sixfold higher than at C_SDS_ = 25.0 mM. This promoted a transition in droplet spreading behavior from partial splashing to uniform spreading, a phenomenon consistent with the observations presented in Figure 5c. The interfacial activity is enhanced by surfactant adsorption at both air/liquid and solid/liquid interfaces, reducing surface tension while improving substrate wettability (Figure 5b). Dynamic surface tension measurements confirm rapid interfacial coverage, reaching≈46.7 mN·m^−1^ at C_SDS_ = 25.0 mM and 43.35 mN·m^−1^ at C_SDS_ = 100 mM within 10 ms, while the equilibrium value drops from 33.69 to 32.50 mN·m^−1^. The negligible change in equilibrium tension suggests near‐saturation adsorption at the interface, while the pronounced reduction in initial tension indicates accelerated adsorption kinetics. This acceleration is attributed to the increased number density of micelles, which rapidly dissociate to replenish monomers at the newly created interface during droplet impact. Collectively, uniform spreading at elevated Reynolds numbers is strictly contingent upon concurrent optimization of both molar ratio and concentration, specifically within the range R_CT4/SDS_ = 10‐12 and 80.0 mM<C_SDS_<150 mM.

The phase diagram presented in Figure S10 systematically correlates droplet impact outcomes with solution viscosity and dynamic surface tension measured at 10 ms. Analysis reveals that droplets exhibiting lower viscosity consistently undergo partial splash upon impacting surfaces. Critically, viscosity exerts a greater influence than dynamic surface tension in governing uniform spreading during impact deposition, with optimal performance requiring both sufficiently high viscous dissipation and rapid interfacial activity. This mechanistic hierarchy demonstrates that while reduced surface tension facilitates initial spreading, sustained spread uniformity depends predominantly on bulk rheological properties that suppress droplet retraction.

The mechanism of the uniform round‐shape spreading by adding CT_4_‐250/SDS25.0 into the impacting water drop is illustrated in Figure 6. For the CT_4_‐250/SDS25.0, because of the reduced charge repulsion and enhanced hydrophobic interaction due to the electrostatic binding between CT_4_ and SDS and resulting in a hydrophobic chain in each surfactant, the surfactant molecules jam at water‐air interface and form entangled wormlike micelle networks in the bulk phase (Figure 6a–c). The three‐dimensional network structure formed by CT_4_/SDS droplets enhanced the system's viscosity and causes a spatial blockage structure composed of highly entangled micellar networks to form at the spreading front, which can maintain the uniformity and stability of the liquid film to resist splashing and spread uniformly during compacting with the cotton fabric (Figure 6d–f). During droplet impingement, CT_4_ rapidly diffuses and dynamically binds SDS molecules at nascent air/water and solid/liquid interfaces through oligomeric self‐assembly to reduce the surface tension and stabilize the new interface until maximum spreading is achieved. Concurrently, CT_4_/SDS aggregates can rapidly migrate to the new interface upon droplet‐substrate collision. The aggregates are jointly formed by CT_4_ and SDS. Coupled with the ionic bonding between CT_4_ and the cellulose backbones, these aggregates effectively inhibit the excessive spreading of droplets during the W‐phase. Consequently, a uniform circular deposition is successfully enabled (Figure 6g–i). From the above phenomena, it can be concluded that the micellar transition from spherical to wormlike structures significantly enhances solution viscosity and alters the kinetics of surface tension reduction, further affecting the droplet spreading phenomenon. Consequently, these optimized conditions collectively induce the following key effects: (1) Formation of a percolating 3D network: Sufficiently elongated worm‐like micelles undergo entanglement, forming a continuous three‐dimensional network that spans the bulk solution. (2) Significant enhancement of bulk viscosity: The system's bulk viscosity increases substantially, reaching values approximately sixfold higher than those of the initial solutions. (3) Promotion of rapid interfacial activity: A concomitant reduction in dynamic surface tension facilitates faster surfactant adsorption and enhanced interfacial activity at newly created surfaces. In conclusion, the resultant microstructure, characterized by pronounced jamming at both air/liquid and solid/liquid interfaces, governs the uniform spread behavior observed during high‐velocity impact.

**FIGURE 6:**
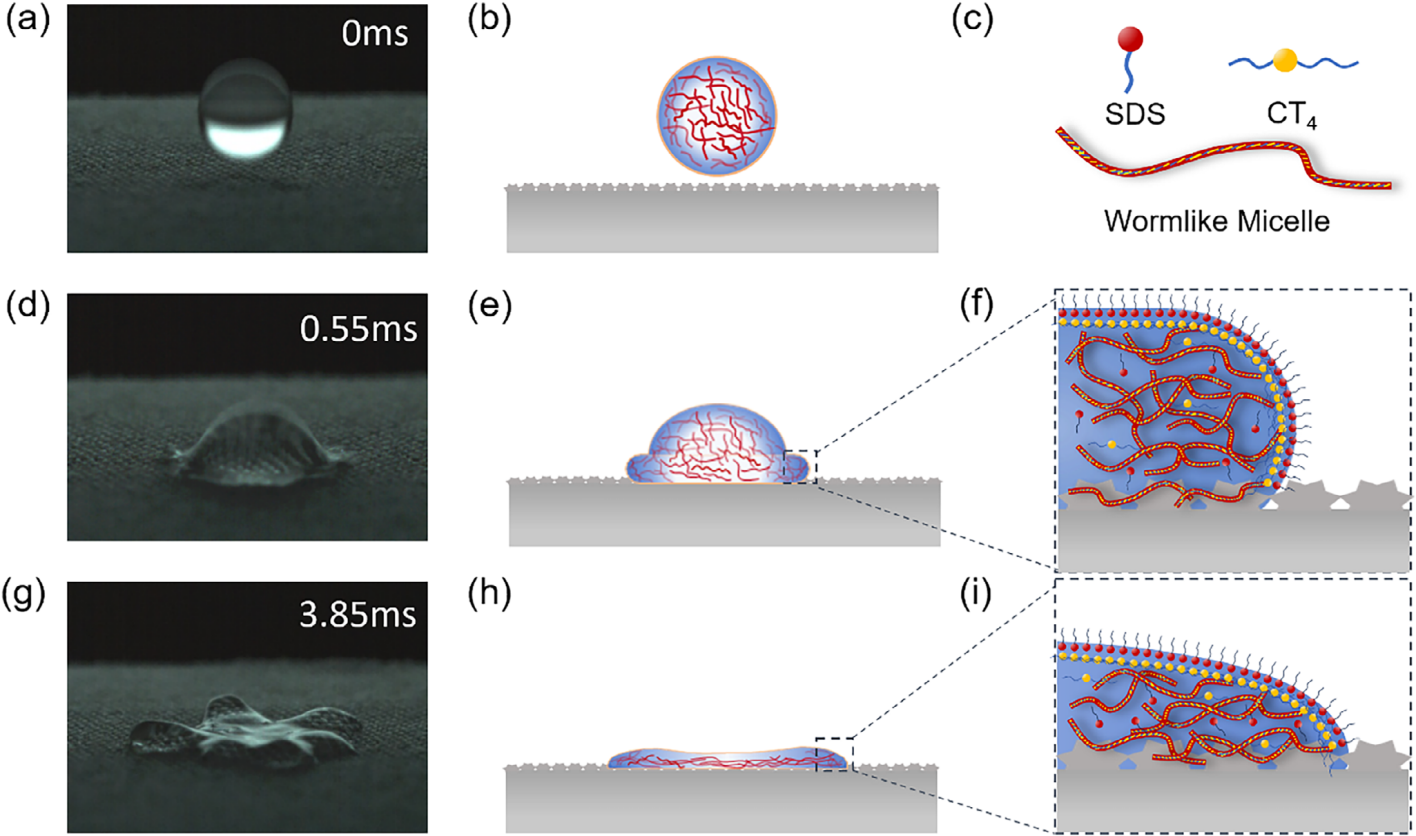
Mechanism of uniform spreading on cotton fabric induced by live‐oligomeric surfactant impact. (a,b) Initial drop containing entangled wormlike micelle networks. (c) Schematic representations of SDS, CT_4_ and wormlike micelles. (d,e) Rapid surfactant diffusion and dense assembly at the advancing water‐air interface, forcing micellar extension and water immobilization. This enables stable, uniform spreading. (f) Strong surfactant‐substrate affinity and entanglement of micellar networks with fabric micro/nanostructures. (g–i) Schematic representations of CT_4_/SDS aggregate inhibiting the diffusion of droplets on the untreated cotton fabrics.

In addition, to further corroborate the specific interaction between CT_4_ and SDS that drives the micellar transition, comparative control experiments were conducted with glycerol, a non‐interacting viscosity modifier. As detailed in Figure S11a,b, the viscosity gradually increases from around 2 to approximately 5 with increasing R_Glycerol/SDS_ ratio. Unlike the CT_4_/SDS system, the increased viscosity here still cause splashing, as shown in Figure S12. In addition, Figure S11c,d shows the addition of glycerol does not cause a significant decrease in dynamic surface tension within 10 ms, whereas the introduction of CT_4_ leads to its rapid reduction, thereby suppressing droplet splashing upon impact on a new interface. This confirms that the uniform spreading observed in the main text is a result of specific CT_4_‐SDS interactions and the resultant wormlike micellar networks, not merely a viscous effect.

The critical factors controlling the uniform spread and deposition of CT_n_/SDS droplets are the electrostatic and hydrophobic interactions between CT_n_ and SDS, as well as the formation of CT_n_/SDS coacervates. As shown in Figure 7a, the electrostatic potential (ESP) mapped onto the van der Waals (vdW) surface and the surface area distribution across ESP ranges [36, 37, 38, 39] are illustrated for SDS and CT_n_, respectively. The vdW surface of SDS exhibits distinct amphiphilic character in its ESP distribution. A region near the sulfuric acid group shows strongly negative ESP values (less than −45.7 kcal mol^−1^), resulting to ion pairing with Na^+^. In contrast, the long alkyl chain, being a hydrocarbon in nature, displays a relatively uniform and symmetric electron density distribution. However, due to the strong electron‐withdrawing effect of the sulfate headgroup, the electron cloud is slightly polarized toward the head, leaving the tail region with a very weakly positive ESP. ESP on the molecular vdW surface exhibits global minima and maxima of −47.0 and +135.1 kcal mol^−1^, corresponding to the oxygen in sulfuric acid group and Na^+^ ion in SDS, respectively. The quaternary ammonium group induces a pronounced positive ESP on both CT_1_ and CT_4_, with values ranging from +43.9 to +124.2 kcal mol^−1^ for CT_1_ and +42.2 to +107.1 kcal mol^−1^ for CT_4_, confirming it as the origin of the maximum positive ESP in each case. Therefore, the ESP analysis indicates that CT_n_ and SDS form stable CT_n_/SDS aggregates through electrostatic attraction between the quaternary ammonium groups on CT_n_ and the anionic sulfonate groups on SDS, coupled with hydrophobic interactions between the alkyl chains of both molecules (Figure 7b). This structural evolution is identified as the determining factor for the uniform spreading and deposition of the resulting droplets. Owing to its longer hydrophobic alkyl chain, CT_4_ exhibits stronger interaction with SDS molecules than CT_1_, as evidenced by their respective binding energies. This enhanced interaction enables CT_4_ to drive the structural transition of SDS micelles from spherical to wormlike at a markedly lower molar ratio (R_CT4/SDS_ = 5). In contrast, CT_1_ requires a much higher molar ratio (R_CT1/SDS_>90) to effect the same transition.

**FIGURE 7:**
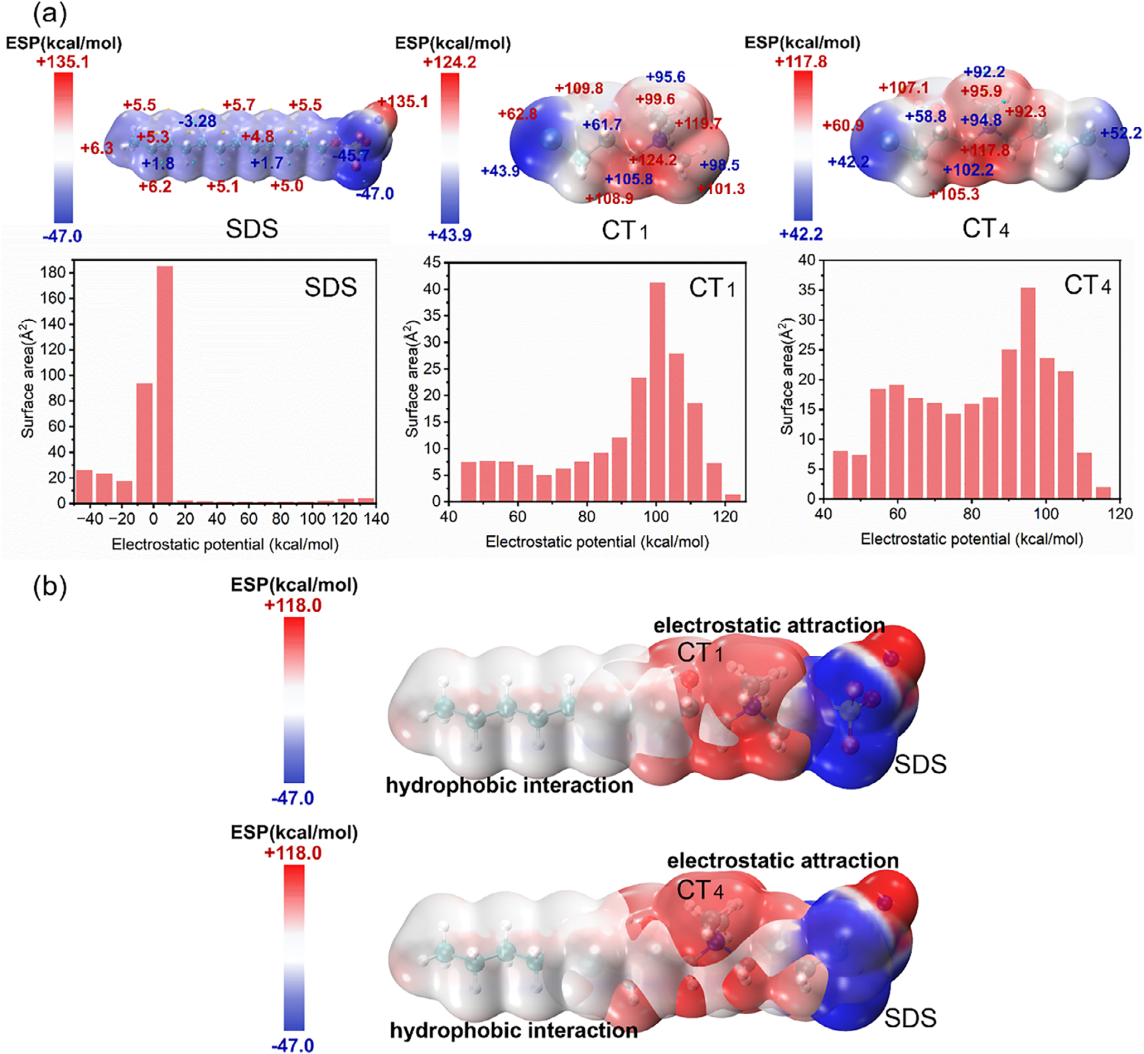
The electrostatic and hydrophobic interactions between CT_n_ and SDS as well as the formation of CT_n_/SDS coacervates. (a) Electrostatic potential (ESP) mapped onto the van der Waals (vdW) surfaces and the corresponding ESP range analysis for SDS and CT_n_. (b) The electrostatic attraction and hydrophobic interaction between SDS and CT_n_.

In summary, three key factors govern the uniform spreading and deposition of CT_n_/SDS droplets. First, electrostatic attraction between the quaternary ammonium groups of CT_n_ and the anionic sulfonate groups of SDS drives the initial co‐assembly. Second, hydrophobic interactions between the alkyl chains of both molecules stabilize the aggregates. Third, the length of the CT_n_ alkyl chain dictates the binding strength and thus the efficiency of micellar structural transition: the longer chain of CT_4_ promotes wormlike micelle formation at a low molar ratio (∼5), whereas CT_1_ requires a much higher ratio (>90) to achieve a similar effect. Collectively, these molecular‐level interactions determine the evolution of surfactant aggregates, which in turn controls droplet spreading dynamics and deposition uniformity.

As illustrated in Figure 8a,b, the reactive dye red 120/CT_4_‐250/SDS25.0 ink demonstrates significantly improved printing precision on cotton fabric compared to the R120/SDS25.0 formulation. When printing lines with nominal widths of 80, 200, and 320 µm, the resulting line widths for the CT_4_‐containing ink measured 119, 280, and 432 µm, respectively, whereas the control R120/SDS25.0 ink produced broader lines of 155, 306, and 461 µm. This corresponds to a reduction in line width of 30.3%, 9.3%, and 6.7% for the respective target widths, confirming the superior confinement capability of the CT_4_‐modified ink (Figure S13a). Similarly, on paper substrates (Figure 8c,d), the reactive dye red 120/CT_4_‐250/SDS25.0 ink exhibited enhanced definition with final widths of 97, 257, and 403 µm for the target line widths, compared to 141, 274, and 419 µm for the R120/SDS25.0 ink. These measurements represent a width reduction of 44.3%, 6.6%, and 4.0% respectively, demonstrating consistent improvement in printing accuracy across different substrates (Figure S13b). This confinement capability is further evidenced by the distinct suppression of droplet splashing for the CT_4_‐containing ink. In contrast, the R120/SDS25.0 ink exhibited more pronounced droplet splashing on paper during impact. The observed improvement in line definition stems from the unique rheological properties of the CT_4_/SDS system.

**FIGURE 8:**
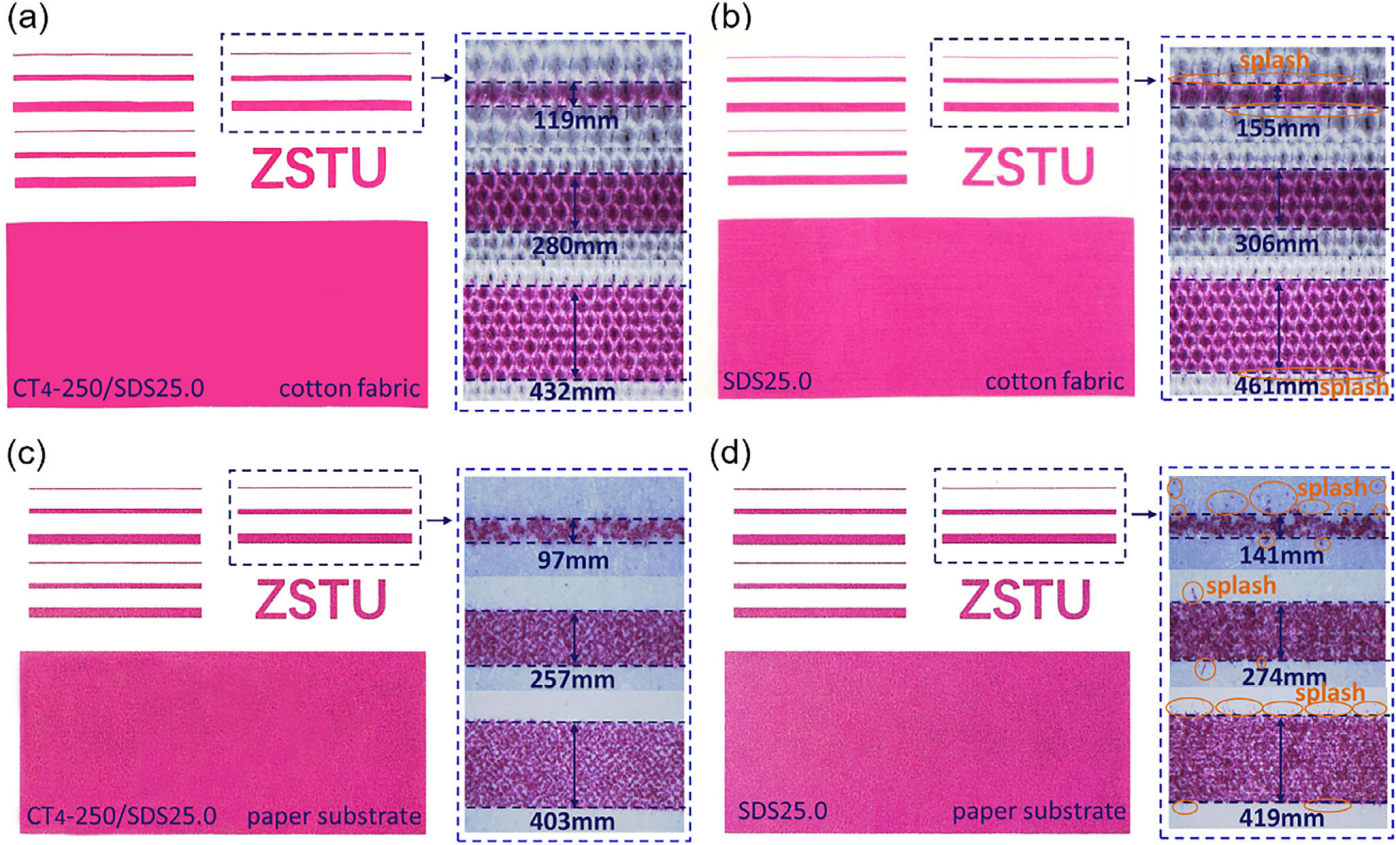
Pattern fineness of reactive dye R120/CT_4_‐250/SDS25.0 and R120/SDS25.0 ink in inkjet printing on cotton fabrics and paper. (a,b) 3D video micrographs of cotton fabric printed with R120/CT_4_‐250/SDS25.0 and R120/SDS25.0 ink. (c,d) 3D video micrographs of paper printed with R120/CT_4_‐250/SDS25.0 and R120/SDS25.0 ink.

In addition, the R120/CT_4_‐250/SDS25.0 ink achieves a K/S value of 3.62, representing a 21.48% enhancement compared to the conventional reactive dye ink (2.98). The significant enhancement in color strength is attributed to the structural transformation of surfactant aggregates, which optimizes the distribution of the dye on the substrate.

This successful application of the R120/CT_4_‐250/SDS25.0 ink in generating well‐defined patterns underscores the critical role of the wormlike micellar network in achieving precise droplet deposition for functional printing, specifically through its dual ability to suppress droplet splashing and to confine excessive spreading while maintaining deposition uniformity.

## 3 Conclusion

This study establishes a sophisticated molecular‐design framework to tailor droplet impact dynamics at a high impact velocity of 1.98 m s^−1^ on cotton fabrics through the programmed self‐assembly of CT_n_/SDS complexes. By modulating the electrostatic and hydrophobic interactions between CT_4_ and SDS, we demonstrate a transition from discrete spherical micelles (∼2.2 nm) to an intricately entangled 3D wormlike micelle (WLM) network. Rheological and structural analyses confirm that the solution viscosity doubles as R_CT4/SDS_ increases from 3 to 10, and markedly rises from 2.85 to 16.92 mPa·s as C_SDS_ scales from 25.0 to 100 mM. Despite a rapid drop in dynamic surface tension to ∼43.35–46.7 mN·m^−1^ within 10 ms, this bulk viscosity enhancement dominates the system to effectively suppress stochastic splashing. Specifically, droplet splashing is fully eliminated when the CT_1_ concentration reaches 2250 mM or when the SDS concentration exceeds 35.0 mM (at a 1:30 ratio). The spreading kinetics are strongly confined: the CT_1_‐4500/SDS150 droplet (Re = 286) exhibits the minimal spreading parameters (α_f_ = 1.81, β_f_ = 3.09), representing a massive restriction compared to pure SDS25.0 (α_f_ = 3.25, β_f_ = 6.87).

The remarkable splash suppression and uniform deposition performance at the optimal composition with an R_CT4/SDS_ of 10–12 and a C_SDS_ ranging from 80.0 to 150 mM stem from a synergistic multi‐scale mechanism. The WLM network establishes a transient viscoelastic surge and a physical spatial barrier at the spreading front. Concurrently, ionic bonding between CT_4_ cations and the cellulose backbone, alongside network entanglement with the fiber micro/nanostructures, induces a strong mechanical pinning effect that immobilizes the three‐phase contact line during the capillary‐driven wetting phase. The practical utility of this strategy is validated via drop‐on‐demand inkjet printing using a reactive dye red 120/CT_4_‐250/SDS25.0 ink formulation. Compared to the control formulation, the CT_4_‐modified ink successfully shrinks printed line widths by up to 30.3% on cotton fabrics and 44.3% on paper, while simultaneously enhancing the color strength (K/S value) by 21.48% (from 2.98 to 3.62).

In conclusion, this work provides a transformative strategy for tailoring high‐speed droplet dynamics via internal structural engineering. This approach thus circumvents the limitations of conventional external‐field‐dependent methods. By bridging the gap between molecular‐scale assembly, transient rheology and macroscopic wetting kinetics, these findings offer a versatile platform for advancing high‐precision technologies. Such technologies include functional inkjet printing, biochip fabrication and targeted pesticide delivery—applications where precise liquid patterning is paramount.

## 4 Experimental Section

### 4.1 Materials

All chemicals and reagents were of analytical grade and used as received unless otherwise stated. *N*,*N*‐Dimethylbutylamine (≥98%), Sodium dodecyl sulfate (SDS) (98.5%), 3‐Chloro‐2‐hydroxypropyl trimethyl ammonium chloride (CT_1_) (60 wt.%) and Glycerol (99%) were obtained from Shanghai Aladdin Biochemical Technology Co., Ltd. (China). Epichlorohydrin was purchased from Shanghai Macklin Biochemical Technology Co., Ltd. (China). HCl (Analytical Reagent) was sourced from Huzhou Shuanglin Chemical Technology Co., Ltd. (China). 3‐Chloro‐2‐hydroxypropyl trimethylbutyl ammonium chloride (CT_4_) (50%) is self‐synthesized in laboratory.

### 4.2 Preparation of Quaternary Ammonium Salt Monomer (CT_4_)

The synthesis methods of the quaternary ammonium salt monomer with 4 alkyl chain length is as follows: the alkyl chain lengths on quaternary ammonium salts is 4, named 3‐Chloro‐2‐hydroxypropyl trimethylbutyl ammonium chloride (CT_4_), was self‐synthesized in laboratory. Deionized water was added to a 250 mL three‐necked round‐bottom flask equipped with a reflux condenser, a magnetic stirrer, and a temperature‐controlled water bath maintained at 5°C using an ice bath. Concentrated hydrochloric acid was then added dropwise via a separatory funnel at a controlled rate. Following complete addition of HCl, 0.1 mol *N*,*N*‐Dimethylbutylamine was charged into the flask, and the mixture was stirred for 1 h at 5°C. Subsequently, the requisite amount of epichlorohydrin was added dropwise via the separatory funnel. Upon complete addition, The experiment was conducted at a temperature of 30°C for a duration of 1 h. The resulting pale yellow liquid was collected and cooled, yielding the target compound, designated CT_4_.

### 4.3 Preparation of CT_n_/SDS Solution

CT_n_/SDS solutions were prepared by dissolving SDS in deionized water, followed by addition of CT_n_ surfactant. Then dissolve to prepare the CT‐m/SDS‐n solution, where m represents the CT concentration (mmol L^−1^) and n represents the SDS concentration (mmol L^−1^). Specifically, for a 20 mL solution of CT_1_‐500/SDS25.0 (denoting 500 mmol L^−1^ CT_1_ and 25.0 mmol L^−1^ SDS), 0.144 g SDS was dissolved in 18.119 mL deionized water under magnetic stirring. Subsequently, 1.881 g CT_1_ was added gradually, and the mixture was stirred magnetically at room temperature for 20 min to ensure complete dissolution and obtain a 500 mM CT_1_/25.0 mM SDS solution, abbreviated as CT_1_‐500/SDS25.0.

### 4.4 Viscosity Measurement

The rheological properties of the solution were determined by assessing the relationship between viscosity and shear frequency. Measurements were conducted using a rotational rheometer (SmartPave 102e, Anton Paar, Austria) at a temperature of 25°C. The instrument converts the measured torque (M) and rotational speed into shear stress (σ) and shear rate (γ̇). The apparent viscosity (η) of the fluid was calculated using the Bingham equation: η = σ/ γ̇.

### 4.5 Modulus Measurement

Dynamic oscillatory tests were conducted using a rotational rheometer (SmartPave 102e, Anton Paar, Austria) to investigate the viscoelasticity of the fluid. In this mode, the fixtures apply a small‐amplitude sinusoidal strain (or stress) to the sample. By analyzing the amplitude and phase shift of the stress response, the storage modulus (G') and loss modulus (G'') are calculated. This allows for the quantitative characterization of the sample's solid‐like (elastic) and fluid‐like (viscous) properties, as well as the strength of its three‐dimensional network structure.

### 4.6 Dynamic Surface Tension Measurement

The dynamic surface tension of the solutions was determined using the maximum bubble pressure method with a dynamic surface tensiometer (BP‐100, KRÜSS, Germany). The capillary tube diameter was first calibrated with the instrument. Subsequently, the surface tension of ultrapure water at 25°C was measured as a baseline to validate the capillary diameter. Following this, the surface tensions of all solutions were measured across a bubble lifetime range of 10 to 10 000 ms at a constant temperature of 25°C.

### 4.7 Particle Size Measurement

The hydrodynamic diameter of solution aggregates was determined by dynamic light scattering (DLS) using a nanoparticle size analyzer (Zetasizer Nano‐S, Malvern Panalytical, UK). The instrument was switched on 30 min in advance to allow for warm‐up and stabilization. After the instrument completed its self‐check and reached a stable state, the prepared solution was loaded into a standard quartz cuvette and placed into the designated slot of the sample chamber. All measurements were conducted at 25°C, and the reported results represent the average of three consecutive measurements.

### 4.8 Small‐Angle X‐Ray Scattering (SAXS) Characterization

SAXS measurements were conducted on a Xeuss 2.0 instrument (Xenocs, France) equipped with a Cu K_α_ X‐ray source (wavelength λ = 1.54189 Å, operating at 30 W) and a Pilatus 3R 300K detector (pixel size: 172 µm). With the sample‐to‐detector distance fixed at 1188 mm, the beam center was calibrated at (x, z) = (236.18, 361.33) for the CT_4_‐200/SDS50.0 sample and (236.35, 361.26) for the SDS50.0 sample. Each sample was exposed for 300 s to obtain two‐dimensional scattering patterns. The raw data were subsequently processed through standard procedures, including background subtraction from the solvent and azimuthal averaging, to obtain the one‐dimensional scattering intensity profile, I(q), as a function of the scattering vector. SAXS is capable of tracking the transition from individual spherical micelles to wormlike micelles.

### 4.9 Cryogenic Transmission Electron Microscopy (Cryo‐TEM) Characterization

The aggregate morphology was characterized using a cryo‐transmission electron microscope (Tecnai F20, FEI, USA). Specifically, 30 µL of the sample solution was deposited onto a copper grid coated with a porous carbon film. Excess liquid was carefully blotted with filter paper to form a thin liquid film, after which the grid was immediately vitrified by rapid immersion into liquid ethane cooled by liquid nitrogen (‐183°C). The vitrified samples were then transferred to a transmission electron microscope (JEOL 1400, Talos, USA) for imaging under 200 kV voltage using low‐dose conditions to minimize beam‐induced damage.

The size of the aggregates observed in cryo‐TEM micrographs was quantified using Nano Measurer software (v1.2.5, Fudan University, China). To ensure statistical reliability of the size distribution analysis, over 150 particles were measured for each experimental condition from multiple, independent images.

### 4.10 Observation of Droplet Spreading

Droplet behavior was captured using a high‐speed imaging system consisting of three primary components: (1) a micropipette tip for generating droplets, with the falling height precisely controlled; (2) a high‐speed camera (Cyclone‐2‐2000‐M/C, Optronis, Germany) operating at an exposure time of 300 µs and a frame rate of 900 fps, was positioned at a 30° angle to the fabric sample stage and maintained at a consistent distance. It was synchronized to trigger simultaneously for capturing the entire process from droplet release, impact, to penetration into the fabric; (3) a LED accent light source providing uniform illumination in backlight configurations, effectively eliminating motion blur by matching the short exposure time of the high‐speed camera. The spreading diameter of droplets on the fabric was quantitatively analyzed using ImageJ software.

### 4.11 Inkjet Printing

The inkjet printing process was performed using a piezoelectric drop‐on‐demand printer (L130, Epson, China). Printing was conducted on two types of substrates: standard 80 g m^−^^2^ A4 paper and cotton fabric. For printing on cotton, fabric specimens were cut to dimensions slightly larger than the printed pattern and firmly adhered to paper substrates before being fed into the printer. A high‐quality color printing mode was selected for all experiments. All printing operations were carried out in an environmentally controlled chamber maintained at 25 ± 1°C and 65 ± 2% relative humidity.

### 4.12 3D Video Microscopy Observation

Each fabric sample was securely mounted on the stage of a 3D video microscope (HIROX KH‐7700, Questar, USA). The focus was then fine‐tuned until a sharp image of the printed pattern was displayed on the monitor. Once a stable and clear image was obtained, it was captured. The widths of lines with different thicknesses were measured, and the images were saved. This procedure was repeated for each subsequent sample. To ensure image clarity and measurement accuracy, appropriate magnification levels were selected according to the line widths: 175x for fine lines, 75x for medium lines, and 50x for coarse lines on CT_n_‐series fabric samples.

## Author Contributions

S.L.L. and L.W. drafted major sections of the manuscript and participated in its revision. L.W. conceived the original concept of this perspective article and designed the article framework. B.B.X., X.Y.L., and Y.D. contributed to writing portions of the text and performing proofreading. J.W.L. and D.M.Q. provided theoretical insights and critical discussion throughout the development of the work.

## Conflicts of Interest

The authors declare no conflicts of interest.

## Supporting information




**Supporting File 1**: advs76684‐sup‐0001‐SuppMat.docx.

**Supporting File 2**: advs76684‐sup‐0002‐MovieS1.mp4

**Supporting File 3**: advs76684‐sup‐0003‐MovieS2.mp4

**Supporting File 4**: advs76684‐sup‐0004‐MovieS3.mp4

**Supporting File 5**: advs76684‐sup‐0005‐MovieS4.mp4

**Supporting File 6**: advs76684‐sup‐0006‐MovieS5.mp4

## Data Availability

The data that support the findings of this study are available from the corresponding authors upon request.
