# Supplementary material for: Regulating Droplet Dynamics via Wormlike Micelle Networks: From Splash Suppression to Precise Confined Spreading
Source: Adv Sci (Weinh). 2026 Jul 29;13(57):e76684. doi: 10.1002/advs.76684 (PMC13418275; doi:10.1002/advs.76684)
Supplement: Supplementary file 1 [file ADVS-13-e76684-s001.docx]

Regulating Droplet Dynamics via Wormlike Micelle Networks: From Splash Suppression to Precise Confined Spreading

Shuling Liu^a^, Beibei Xie^a^, Xingyue Lou^a^, Yu Deng^a^, Lei Wang^a^*, Jiawei Li^abc^, Dongming Qi^abc^

^a^ *Engineering Research Center for Eco-Dyeing and Finishing of Textiles, Zhejiang Sci-Tech University, Hangzhou 310018, Zhejiang, China*

^b^ *Key Laboratory of Advanced Textile Materials and Manufacturing Technology and*

*Engineering Research Center for Eco-Dyeing & Finishing of Textiles, Ministry of*

*Education, Zhejiang Sci-Tech University, Hangzhou 310018, Zhejiang, China*

^c^ *Zhejiang Provincial Innovation Center of Advanced Textile Technology, Shaoxing*

*312000, Zhejiang, China*

*Corresponding Author: Lei Wang;

Email: [wanglei90@zstu.edu.cn](mailto:wanglei90@zstu.edu.cn);

Figure S1. Dynamical behavior of impacting SDS and CT_4_/SDS25.0 drops with different CT_4_ concentrations on the cotton fabric.

Figure S2. Dynamical behavior of impacting CT_4_/SDS drops with different SDS concentrations on the cotton fabric.

Figure S3. The maximum spreading parameter α_f_ during K-phase for different CT_n_/SDS system.

Figure S4. The final spreading parameter β_f_ for different CT_n_/SDS system.

Figure S5. The particle size of CT_4_/SDS25.0. At a fixed C_SDS_ of 25.0 mM with no CT_4_ (R_CT4/SDS_=0), the results of Cryo-TEM showed the formation of spherical micelles approximately 2.2 nanometers in size, which was consistent with the results of the DLS measurement.

Figure S6. Scattering intensity at different R_CT4/SDS_ when the C_SDS_ is fixed at 50.0 mM. (a) Scattering intensity when R_CT4/SDS_=0 at C_SDS=_50.0 mM, no significant intensity variation at low q region indicates that the polymer type is spherical. (b) Scattering intensity when R_CT4/SDS_=4 at C_SDS=_50.0 mM, the variation trends is significantly different from the trend of the samples with R_CT4/SDS_=0 at C_SDS=_50.0 mM at low q region, indicating a significant change in the type of aggregates. The aggregate structure transforms from spherical micelles into wormlike micelles.

Figure S7. The influence of R_CT1/SDS_ molar ratio on the viscosity of CT_1_/SDS system with fixed SDS at 25.0 mM.

Figure S8. The influence of SDS concentration on the viscosity of the CT_1_/SDS system when the molar ratio of fixed R_CT1/SDS_ is 30.

Figure S9. Variation of storage modulus (G') and loss modulus (G'') as a function of oscillatory shear frequency for the CT_n_/SDS system_._ G' is always less than G'', indicating that the CT_n_/SDS surfactant shows a liquid-like Newtonian flow behavior.

Figure S10. The phase diagram of impacting phenomena with dynamic surface tension and viscosity of CT_1_/SDS and CT_4_/SDS.

Figure S11. (a) The viscosity of Glycerol/SDS with fixed SDS at 25.0 mM. (b) The viscosity of Glycerol/SDS with fixed SDS at 32.5 mM. (c) Dynamic surface tension of Glycerol/SDS with fixed SDS at 25.0 mM. (d) Dynamic surface tension of Glycerol/SDS with fixed SDS at 32.5 mM. As shown in Figure S11a and S11b, the viscosity gradually increases from around 2 to approximately 5 with increasing R_Glycerol/SDS_ ratio. In contrast, Figure S11c and S11d show that the dynamic surface tension profiles, including both the initial and equilibrium values, remain largely unchanged as the ratio of R_CT4/SDS_ increases.

Figure S12. (a) Dynamical behavior of impacting Glycerol/SDS25.0 drops with different Glycerol concentrations on the cotton fabric. (b) Dynamical behavior of impacting Glycerol/SDS32.5 drops with different Glycerol concentrations on the cotton fabric. While the addition of glycerol proportionally increased the bulk viscosity (to 5.45 mPa·s) of the SDS solution (Figure S11a, S11b), it failed to induce any morphological change in the SDS micellar structure. Consequently, the impact dynamics of glycerol/SDS droplets (Figure S12a, S12b) exhibited only heightened splashing due to increased viscous dissipation, never achieving the uniform spreading characteristic. Conversely, a similar viscosity increase within the R_CT4/SDS_ range of 3-10 effectively suppressed splashing and promoted uniform spreading (Movie S5). This stark contrast between Glycerol/SDS and CT_4_/SDS unequivocally demonstrates that the observed suppression of splashing and transition to uniform spreading is not a mere consequence of increased viscosity, but is uniquely contingent upon the oligomeric network formation enabled by the electrostatic binding and hydrophobic interactions between CT_4_ and SDS.

Figure S13. Average widths of coarse, medium and fine lines printed with R120/CT_4_-250/SDS25.0 as well as R120/SDS25.0 ink in inkjet printing on (a) cotton fabric and (b) paper.

Table S1. The viscosity, surface tension, and Re parameters of different types of SDS/CT_n_

|  | Viscosity (cP) | surface tension (mN/m) | Re |
| --- | --- | --- | --- |
| CT_1_-500/SDS25.0 | 3.28 | 54.5 | 2201 |
| CT_1_-750/SDS25.0 | 4.55 | 57.0 | 1587 |
| CT_1_-1000/SDS25.0 | 4.67 | 59.6 | 1553 |
| CT_1_-1250/SDS25.0 | 4.88 | 60.3 | 1484 |
| CT_1_-1500/SDS25.0 | 5.05 | 61.5 | 1443 |
| CT_1_-2250/SDS25.0 | 5.16 | 63.2 | 1407 |
| CT_1_-825/SDS27.5 | 4.43 | 57.5 | 1630 |
| CT_1_-900/SDS30.0 | 4.32 | 58.3 | 1679 |
| CT_1_-975/SDS32.5 | 4.25 | 57.4 | 1704 |
| CT_1_-1050/SDS35.0 | 4.61 | 57.0 | 1581 |
| CT_1_-1500/SDS50.0 | 6.86 | 57.7 | 1058 |
| CT_1_-3000/SDS100 | 16.51 | 60.2 | 438 |
| CT_1_-4500/SDS150 | 25.21 | 63.1 | 286 |
| CT_4_-75.0/SDS25.0 | 2.46 | 43.7 | 2943 |
| CT_4_-100/SDS25.0 | 2.85 | 46.7 | 2552 |
| CT_4_-125/SDS25.0 | 4.45 | 47.3 | 1632 |
| CT_4_-175/SDS25.0 | 4.64 | 48.5 | 1561 |
| CT_4_-250/SDS25.0 | 4.90 | 48.9 | 1480 |
| CT_4_-375/SDS25.0 | 3.98 | 49.2 | 1826 |
| CT_4_-500/SDS25.0 | 3.38 | 49.5 | 2142 |
| CT_4_-110/SDS27.5 | 2.98 | 45.7 | 2441 |
| CT_4_-120/SDS30.0 | 4.01 | 47.1 | 1811 |
| CT_4_-130/SDS32.5 | 4.24 | 45.6 | 1708 |
| CT_4_-140/SDS35.0 | 5.51 | 45.2 | 1316 |
| CT_4_-200/SDS50.0 | 11.18 | 45.2 | 650 |
| CT_4_-400/SDS100 | 16.92 | 43.4 | 428 |
| CT_4_-600/SDS150/ | 14.23 | 43.0 | 507 |
